# [Retracted] Effects of TLR4 gene silencing on the proliferation and apotosis of hepatocarcinoma HEPG2 cells

**DOI:** 10.3892/ol.2025.15187

**Published:** 2025-07-14

**Authors:** Yating Liu, Tao Li, Yuanhong Xu, Enjun Xu, Min Zhou, Baolong Wang, Jilong Shen

Oncol Lett 11: 3054–3060, 2016; DOI: 10.3892/ol.2016.4338

Following the publication of the above paper, it was drawn to the Editor's attention by a concerned reader that, regarding the flow cytometric plots shown in Fig. 4 on p. 3058, the ‘Blank’ and ‘Lipo’ panels (featured on the right) appeared to show similar groupings of dots, which would not have been anticipated if these experiments had been performed discretely under different experimental conditions, suggesting a fundamental flaw either in the way in which these experiments were performed, or in how the results were outputted.

The Editor of *Oncology Letters* has decided that this paper should be retracted from the Journal on account of a lack of confidence in the presented data. The authors were asked for an explanation to account for these concerns, but the Editorial Office did not receive a satisfactory reply. The Editor apologizes to the readership for any inconvenience caused.

